# The Editor's Letter-Box

**Published:** 1916-07-22

**Authors:** H. Willoughby Lyle

**Affiliations:** Dean. King's College Hospital Medical School (University of London). King's College Hospital, Denmark Hill, London, S.E.


### THE EDITOR'S LETTER-BOX.

### The Hospitals and Women Students.

*To the Editor of* The Hospital.

Sir,—On page 362 I observe the following: "Hard on the heroes of King's College Hospital, etc." I write to point out a mistake.

At present women students are not admitted to the medical school of King's College Hospital for final studies. I shall be obliged if you will correct the mistake in your next issue.—Yours faithfully,

H. Willoughby Lyle, Dean.
King's College Hospital Medical School
(University of London).

King's College Hospital, Denmark Hill,
London, S.E., July 17, 1916.

